# Preexisting Cardiovascular Risk Factors and Coronary Artery Atherosclerosis in Patients with and without Cancer

**DOI:** 10.1155/2022/4570926

**Published:** 2022-02-01

**Authors:** Junyi Guo, Peng Fang, Wei Shi, Pengcheng Luo, Shengqi Huo, Dan Yan, Moran Wang, Dewei Peng, Lintong Men, Sheng Li, Jiagao Lv, Li Lin

**Affiliations:** ^1^Division of Cardiology, Department of Internal Medicine, Tongji Hospital, Tongji Medical College, Huazhong University of Science and Technology, Wuhan, China; ^2^Division of Cardiology, Department of Internal Medicine, The Fifth Hospital of Huangshi, Huangshi, China; ^3^Department of Geriatrics, Tongji Hospital, Tongji Medical College, Huazhong University of Science and Technology, Wuhan, China

## Abstract

Cancer survivors suffer a higher risk of coronary artery atherosclerosis (CAA). Whether cancer patients had increased baseline CAA burden prior to cardiotoxic therapy remains unclear. We conducted a case-control study, and 286 consecutive patients were finally included. Among these patients, 181 had newly diagnosed cancer and 105 had nonmalignant diseases. Cancer was confirmed by biopsy. The severity of CAA was determined by coronary angiography and evaluated using the percentage of stenosis or Gensini scoring (GS). Patients with cancer versus cancer-free controls were older (OR = 1.052, 95% CI: 1.021–1.084, *p* < 0.001), more commonly male (OR = 0.048, 95% CI: 1.004–2.676, *p*=0.048), and more severely exposed to smoking (OR = 1.020, 95% CI: 1.007–1.033, *p*=0.003). Cancer patients were significantly more commonly complicated by ≥90% coronary stenosis than the gender- and age-matched cancer-free controls (9/93 versus 1/93, OR = 4.875, 95% CI: 1.024–23.213, *p*=0.047). After adjustment for age, gender, hypertension, diabetes, smoking history, blood glucose, and total cholesterol, cancer was significantly associated with high GS (adjusted OR = 2.208, 95% CI: 1.077–4.524, *p*=0.031). Our study demonstrated that cancer patients had increased CAA burden prior to cardiotoxic therapy. Further study is necessary to investigate the link between CAA and cancer.

## 1. Introduction

Cardiovascular disease (CVD) and cancer are the leading causes of death worldwide with utterly different treatment strategies [1, 2]. In recent years, emerging evidence suggests a relation between the two seemingly disconnected diseases [3]. Survivors of cancer suffer a higher risk of CVD [4–7], while cohort studies also found that patients with CVD are more likely to develop cancer [8–10]. However, how the two appealingly separated diseases are linked is not clear.

Oncocardiology is a new field of clinical medicine that addresses the overlap between cancer and CVD [11]. During the decade of research of oncocardiology, the cardiotoxicity of the chemo-, radio-, and immunotherapy of cancer remains the principal focus [5, 12–14]. Accumulating evidence has shown that cardiovascular injury caused by cancer therapy is associated with poor prognosis, especially in patients with preexisting cardiovascular risk factors and cardiovascular diseases [15, 16]. Adding to the challenge is the fact that preexisting cardiovascular diseases in cancer patients are quite common and many patients with cancer show cardiac injury even before cardiotoxic treatments [7, 17, 18]. In 2015, S. G. Al-Kindi et al. first found that cancer patients had higher prevalence of preexisting CVD than the age-matched general population [7]. However, this finding was not the major content of their study, and to our knowledge, there were no other studies confirming this finding since then. Whether cancer patients had increased CVD burden prior to cardiotoxic therapy and the reason behind remain unclear.

It is currently assumed that the shared risk factors of CVD and cancer play a role in the association between the two diseases [19–21]. Actually, many risk factors, such as smoking and diabetes, may cause CVD and cancer at the same time. This might partly explain the increased burden of preexistence of CVD in cancer patients. However, currently, almost all related studies are focused on cancer survivors [22, 23]. Studies evaluating the cardiovascular risk factors and CVD burden in cancer patients before active treatment are lacking.

Coronary artery atherosclerosis (CAA) is one of the most common CVDs. Here, we undertook a case-control study to investigate the distribution of preexisting cardiovascular risk factors and severity of CAA in patients with cancer and cancer-free controls. We aimed to compare the severity of CAA between cancer patients and cancer-free controls and provide clues for further research to investigate the reason behind the increased preexisting CVD burden in cancer patients.

## 2. Materials and Methods

### 2.1. Data Source and Study Design

**Figure 1:**
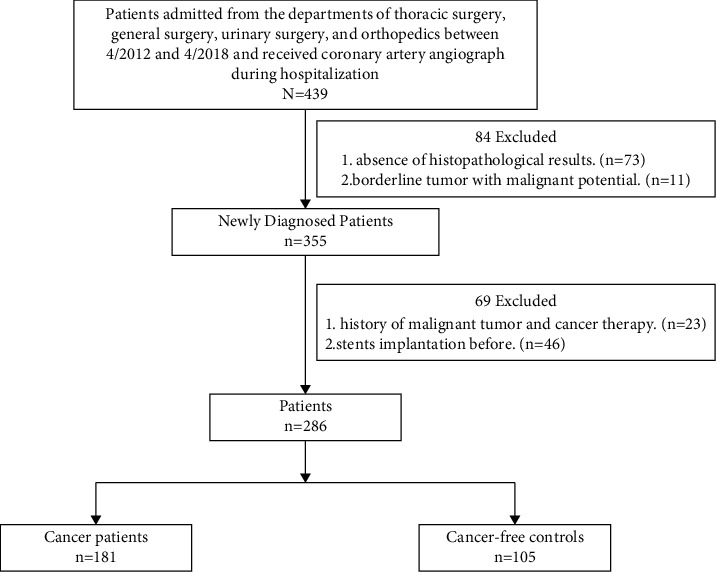
Patient inclusion and exclusion details.

We performed a real-world database analysis complied with the Declaration of Helsinki, and it was approved by the hospital's ethical review board (Tongji Hospital, Huazhong University of Science and Technology, Wuhan, China). Written informed consent was not obtained because the data were analyzed retrospectively and anonymously. Potential eligible patients were identified by screening lists of admissions from the departments of thoracic surgery, general surgery, urinary surgery, and orthopedics of Tongji Hospital between 4/2012 and 4/2018. The majority of the patients were found lung, liver, gastrointestinal, or urinary mass and admitted to hospital for suspected cancer. Initially included in the study were 439 consecutive patients who received coronary artery angiograph during hospitalization. While most patients received established diagnoses by pathologic examination, several patients had indeterminate lesions. In total, 84 patients were excluded for indefinite pathological diagnosis. Among these patients, 73 patients were excluded for the absence of histopathological results to identify the nature of the mass. Eleven patients were excluded because they had borderline tumor with malignant potential. Other exclusion criteria included history of cancer therapy (*n* = 23) and stent implantation (because the existence of stents made us unable to make accurate evaluation of the real stenosis, *n* = 46). Our final study included 286 patients, among whom 181 were diagnosed with cancer and 105 were cancer free (Figure 1).

### 2.2. Definition of Risk Factors

Data were retrieved from the medical records and electronic databases of Tongji Hospital. History of hypertension and diabetes was reported by patients themselves, taken by their residents, and recorded in patients' hospital medical records. The self-reported hypertension/diabetes was defined by answering “yes” to the question “Do you have hypertension/diabetes?” The history of smoking was defined as having consumed tobacco at least once in the past years and was quantified by the smoking index, which was calculated by multiplying the number of packs of cigarettes smoked per day by the number of years the person has smoked.

### 2.3. Coronary Angiography and Gensini Score Assessment

Coronary angiographies were performed using transradial or transfemoral approaches by experienced cardiologists. The extent of coronary stenosis was evaluated by at least two independent physicians, and a final written report was signed after discussion. The Gensini score was calculated according to the previously published method [24]. Briefly, reduction in lumen diameter was evaluated as different scores, and each vascular segment was weighed by a different coefficient. Reductions of 25%, 50%, 75%, 90%, and 99% and complete occlusion were evaluated as 1, 2, 4, 8, 16, and 32, respectively. The left main coronary artery was weighed by 5, proximal segment of the left anterior descending (LAD) coronary artery and the proximal segment of the circumflex artery by 2.5, the midsegment of the LAD by 1.5, the right coronary artery, the distal segment of the LAD, the middistal region of the circumflex artery, the posterolateral artery, and the obtuse marginal artery by 1.0, and other segments by 0.5. The enrolled patients were classified into the two groups (low group 0–18 points; high group >18 points).

### 2.4. Statistical Analyses

The data were analyzed by SPSS version 24.0 for Mac (SPSS Inc., Chicago, IL, USA). Continuous variables with normal distribution were presented as mean ± SD. Nonnormal variables were reported as median (Q1–Q3 quartiles). The normality of distribution of continuous variables was tested by the one-sample Kolmogorov–Smirnov test. Categorical variables were expressed as number (percentage). Means of 2 continuous normally distributed variables were compared by independent-sample Student's *t*-test. The Mann–Whitney U test was run to determine if there were differences between two nonnormal variables. The frequencies of categorical variables were compared using Pearson *χ*^2^ (with or without continuity correction) or Fisher's exact test, when appropriate. Some covariates (smoking index, total cholesterol, and blood sugar) had missing values, and we applied max likelihood by expectation maximization and built imputation data to replace the missing values. Since there existed an imbalance in gender and age between patients in the malignant group and nonmalignant group, we used the case-control matching method and sampled 186 patients from the 286 patients. The binary logistic regression analysis was used to estimate the odds ratios (ORs) and 95% confidence intervals (CIs) for the association between cancer and severity of coronary artery atherosclerosis. A *p* value of <0.05 was set as the level of statistical significance.

## 3. Results

### 3.1. Clinical Characteristics in Patients with Malignant and Nonmalignant Diseases

A total of 286 patients were finally included in this study. To assess the distribution of conventional CVD risk factors in cancer patients and their cancer-free controls, patients were classified into two groups based on their pathological examination results. It turned out that 181 (65.2%) patients had cancer, while 105 (53.3%) patients were cancer free. Among the cancer patients, 83 patients had lung cancer, 41 had esophagus cancer, 18 had colorectal cancer, 12 had gastric cancer, 6 had kidney cancer, 6 had hepatobiliary cancer, 5 had bladder cancer, and 10 had other cancers (Supplementary Figure 1a). According to the TNM classification system of the International Union Against Cancer (8^th^ edition), 68 patients had stage I cancer, 48 stage II, 40 stage III, 8 stage IV, and 25 patients had unknown stage (Supplementary Figure 1b). For the total 286 patients, the median age was 64 years (59–69), including 174 (60.8%) men. Patients with cancer were older (OR = 1.052, 95% CI: 1.021–1.084, *p* < 0.001), more commonly male (OR = 0.048, 95% CI: 1.004–2.676, *p*=0.048), and more severely exposed to smoking (OR = 1.020, 95% CI: 1.007–1.033, *p*=0.003). There were no significant differences in the history of hypertension (OR = 1.379, 95% CI: 0.849–2.236, *p*=0.194) and diabetes (OR = 0.572, 95% CI: 0.281–1.165, *p*=0.124) between the two groups. Blood sugar (OR = 1.066, 95% CI: 0.892–1.272, *p*=0.661) and total cholesterol (OR = 1.081, 95% CI: 0.827–1.413, *p*=0.575) showed no significant differences between the patients with and without cancer (Table 1).

### 3.2. Severity of CAA in Patients with Malignant and Nonmalignant Diseases

**Figure 2:**
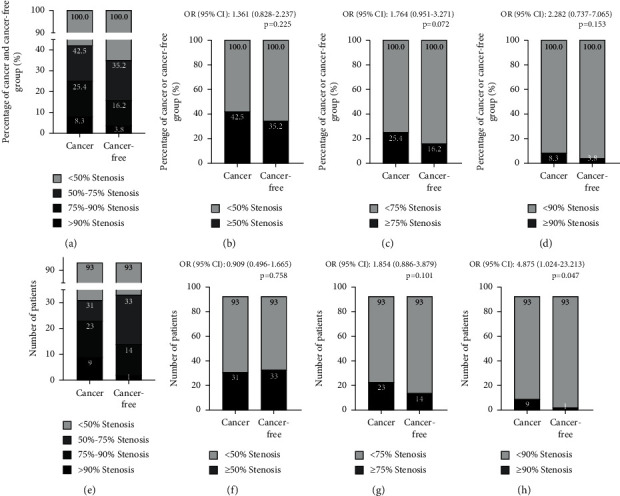
Severity of CAA in cancer and no-cancer patients. (a–d) Comparison of severity of CAA between cancer and no-cancer patients. (a) Cancer patients were more likely to have worse CAA compared with the cancer-free controls. (b), (c), (d) Cancer patients were slightly more likely to have ≥50%, ≥75%, and ≥90% coronary stenosis compared with the cancer-free controls. (e–h) Comparison of severity of CAA between gender- and age-matched cancer and no-cancer group. (h) Patients in the cancer group were significantly more commonly complicated by ≥90% coronary stenosis than patients in the no-cancer group (9/93 versus 1/93, OR = 4.875, 95% CI: 1.024–23.213, *p*=0.047).

We next evaluated the incidence and severity of CAA in the both groups (Figure 2(a)). As shown in Figure 2(b), 42% of the cancer patients and 35.2% of cancer-free controls turned out to have ≥50% coronary stenosis. More cancer patients had ≥75% coronary stenosis compared to patients with no cancer (25.4% versus 16.2%, Figure 2(c)). Also, more cancer patients had ≥90% coronary stenosis compared to the cancer-free patients (8.3% versus 3.8%, Figure 2(d)). There was significant difference in age and gender between the two groups. Using Case-Control Matching function of SPSS, 93 cancer patients were gender and age (±2 years old) matched by 93 cancer-free controls. Basic characteristics of the gender- and age-matched 186 patients are presented in Table 2. The average age was 62 ± 8 years, and 96 (52%) were male. There were no significant differences in all common risk factors. Slightly fewer cancer patients had ≥50% coronary stenosis compared to the cancer-free controls (31/93 versus 33/93, OR = 0.909, 95% CI: 0.496–1.665, *p*=0.758). As shown in Figure 2(g), 23/93 cancer patients and 14/93 of patients with no cancer had ≥75% stenosis (OR = 1.854, 95% CI: 0.886–3.879, *p*=0.101). Notably, cancer patients were significantly more commonly complicated by ≥90% coronary stenosis than the cancer-free controls (9/93 versus 1/93, OR = 4.875, 95% CI: 1.024–23.213, *p*=0.047, Figure 2(h)).

### 3.3. Association between Cancer and Worse CAA

**Figure 3:**
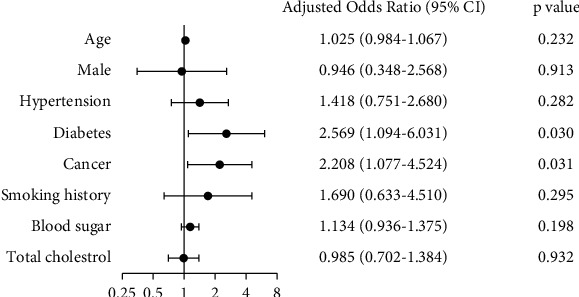
Forest plot of the multivariable logistic regression model. Cancer is significantly associated with high Gensini score (adjusted OR = 2.208, 95% CI: 1.077–4.524, *p*=0.031). CI indicates confidential interval.

Since age, gender, hypertension, diabetes, smoking history, blood glucose, and total cholesterol are all significant long-term risk factor for CAA [19], to verify whether or not there are correlations between cancer and worse CAA besides the shared risk factor association, a binary logistic regression analysis was then performed using the entry process. Variables included in the model were age, gender, hypertension, diabetes, smoking history, blood glucose, total cholesterol, and cancer. As shown in Figure 3, after adjusting for other risk factors, cancer was found to be still significantly associated with worse CAA (adjusted OR = 2.208, 95% CI: 1.077–4.524, *p*=0.031).

## 4. Discussion

In this case-control study, we assessed the distribution of preexisting cardiovascular risk factors and severity of CAA in 286 consecutive patients with or without cancer. We found patients with cancer versus cancer-free controls were significantly older, more commonly male, and more severely exposed to smoking. Cancer patients were more likely to have worse CAA compared to the gender- and age-matched cancer-free controls before active treatment. Multivariate analyses revealed that, after adjustment for age, gender, hypertension, diabetes, smoking history, blood glucose, and total cholesterol, cancer was significantly associated with worse atherosclerosis.

Advances in medical therapies and technologies have prolonged the survival time of patients with cancer and increased the overlap between cancer and CVD. Several previous cohort studies have shown that cancer survivors have increased risk for CAA compared to the general population [22, 25]. However, to our knowledge, few studies ever estimated the baseline CAA burden in cancer patients before active treatment. We found that cancer patients were more likely to have worse CAA even before cardiotoxic cancer treatment, which should draw more attention from oncologists, since many antitumor treatments, such as fluoropyridines, cisplatin, nilotinib, VEGF inhibitors, and radiotherapy, may accelerate coronary artery atherosclerosis or plaque rapture [12, 26]. This result is consistent with the prior findings that preexisting CVDs (CAA, carotid artery disease, peripheral vascular disease, cerebrovascular disease, and heart failure) are more common in untreated cancer patients than the gender- and age-matched general population [7]. However, different from the previous study, our research focused on CAA and tried to further assess the distribution of cardiovascular risk factors between the two groups.

CAA and cancer possess several similar risk factors, which should lead to the concurrence of CAA and cancer in the same individuals [19]. However, most of the existing studies are focused on cancer-free patients or cancer survivors. We compared the distribution of cardiovascular risk factors in newly diagnosed patients and no-cancer controls and found that cancer patients were significantly older, more commonly male, and more severely exposed to smoking. This finding might partly explain the previous result that cancer patients were also more likely to have worse CAA even before cardiotoxic cancer treatments.

In our gender- and age-matched model, the distribution of common CAA risk factors showed no significant difference between the cancer and no-cancer group after the match, but the extent of CAA was still worse in cancer patients. Our multivariate analysis also demonstrated that cancer was associated with worse atherosclerosis after adjustment for some of the common CVD risk factors. This may be explained by that there are still many other risk factors which were not accounted for by the study. On the other hand, it may also suggest that the correlation between cancer and CAA can be pathophysiological [19–21]. There are many shared molecular factors critical to CAA and cancer. For example, chronic inflammation is a common cause for both atherosclerosis [27] and cancer [28]. Besides, it is interesting to mention that an analysis of the Canakinumab Anti-inflammatory Thrombosis Outcomes Study (CANTOS) showed that selective inhibition of interleukin-1*β* with canakinumab decreased the rate of recurrent cardiovascular events and showed its most pronounced effect on reducing lung cancer mortality at the same time [29].

There are several inevitable limitations in our study. First, the case-control design of the study inherently limits our ability to make causal conclusions about the findings. Second, the small sample size limits the generalizability of our conclusions. Moreover, despite our efforts to adjust for many available confound factors, we did not assess the current use of cardiovascular medications and some other confounding factors of interest to clinicians, such as low-density lipoprotein and body mass index, due to the lack of clinic data. We hope to expand the sample size and try to collect multicenter data to obtain more reliable achievements in future.

In summary, our study demonstrated that cancer patients were more likely to have worse CAA before active treatment compared to the general population, which should draw attention from clinicians. A new strategy targeting the shared risk factors and the potential shared pathophysiological process may have synergistic benefits in the prevention and treatment of both CAA and cancer.

## 5. Conclusions

Cancer patients have a heavier baseline CAA burden than cancer-free controls before active cancer treatment. Further study is necessary to investigate the reason behind the increased preexisting CAA burden in cancer patients.

## Figures and Tables

**Table 1:** Basic characteristics of patients included.

Parameter	All patients, *n* = 286	Cancer patients, *n* = 181	Cancer-free controls, *n* = 105	OR (95% CI) for cancer	*P* value
Age	64 (59, 69)	65 (61, 70)	61 ± 10	1.052 (1.021–1.084)	＜0.001
Male	174 (60.8%)	118 (65.2%)	56 (53.3%)	1.639 (1.004–2.676)	0.048
Hypertension	137 (47.9%)	92 (50.8%)	45 (42.9%)	1.378 (0.849–2.236)	0.194
Diabetes	35 (12.2%)	18 (9.9%)	17 (16.2%)	0.572 (0.281–1.165)	0.124
Smoking history/bag-year	12 (0, 27)	17 (0, 30)	0 (0, 17)	1.020 (1.007–1.033)	0.003
Blood sugar/mmol/L	5.35 (4.92, 5.94)	5.41 (4.97, 5.95)	5.34 (4.81, 5.97)	1.066 (0.892–1.272)	0.661
Total cholesterol/mmol/L	4.02 ± 0.91	4.05 ± 0.89	3.98 ± 0.95	1.081 (0.827–1.413)	0.575

**Table 2:** Basic characteristics of patients matched by gender and age.

Parameter	All patients, *n* = 186	Cancer patients, *n* = 93	Cancer-free controls, *n* = 93	OR (95% CI) for cancer	*P* value
Age	62 ± 8	63 ± 7	62 ± 8	1.007 (0.970,1.047)	0.704
Male	96 (52%)	48 (52%)	48 (52%)	1.000 (0.563,1.777)	1.000
Hypertension	103 (55%)	51 (55%)	52 (56%)	0.649 (0.364,1.158)	0.143
Diabetes	24 (13%)	9 (10%)	15 (16%)	1.795 (0.743,4.336)	0.194
Smoking history/bag-year	0 (0, 20)	0 (0,30)	0 (0,17)	1.013 (1.000,1.027)	0.051
Blood sugar/mmol/L	5.41 (4.99,6.11)	5.47 (5.07,6.16)	5.35 (4.86,6.08)	1.098 (0.882,1.367)	0.402
Total cholesterol/mmol/L	4.03 ± 0.95	4.02 ± 0.95	4.03 ± 0.95	0.977 (0.717,1.331)	0.884

## Data Availability

The data used to support the findings of this study are available from the corresponding author upon request.

## Supplementary Materials

Supplementary Materials Supplementary Figure 1: cancer type and cancer stage of patients. (a) Pie chart showing the cancer type distribution of patients. (b) Pie chart showing the cancer stage distribution of patients.
