# Supplementary material for: Preexisting Cardiovascular Risk Factors and Coronary Artery Atherosclerosis in Patients with and without Cancer
Source: Cardiol Res Pract. 2022 Feb 1;2022:4570926. doi: 10.1155/2022/4570926 (PMC8826118; doi:10.1155/2022/4570926)
Supplement: Supplementary Materials — Supplementary Figure 1: cancer type and cancer stage of patients. (a) Pie chart showing the cancer type distribution of patients. (b) Pie chart showing the cancer stage distribution of patients. [file 4570926.f1.docx]

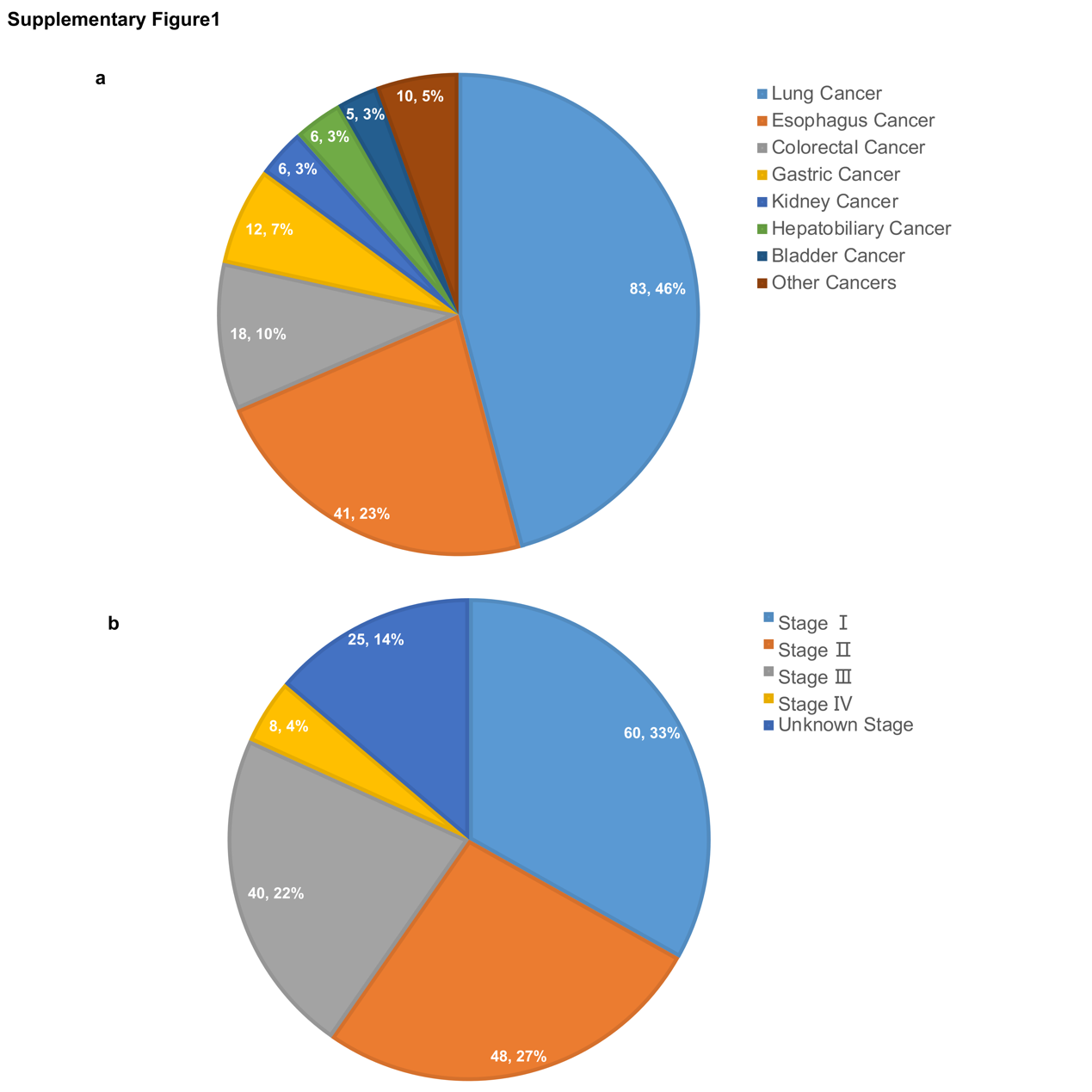


Supplementary Figure1: Cancer type and cancer stage of patients. (a) Pie chart showing the cancer type distribution of patients. (b) Pie chart showing the cancer stage distribution of patients.
